# The Interaction of Immune Priming with Different Modes of Disease Transmission

**DOI:** 10.3389/fmicb.2016.01102

**Published:** 2016-07-13

**Authors:** Ann T. Tate

**Affiliations:** Department of Biology and Biochemistry, University of HoustonHouston, TX, USA

**Keywords:** immune priming, obligate killer, direct transmission, life history tradeoffs, infectiousness

Immune priming in invertebrates is most commonly described as an increase in survival (Roth et al., 2010) or the strength of an immune response (Moret, 2006) against a microbe to which the host has been previously exposed. Because priming alters epidemiologically relevant parameters like disease-induced mortality and recovery, priming is likely to impact the spread, and persistence of diseases in invertebrate populations. Early modeling efforts have explicitly incorporated priming into disease transmission frameworks by allowing exposed (Tidbury et al., 2012) or previously infected but recovered (Tate and Rudolf, 2012) individuals to transition into a primed compartment. There, they are less likely to become infected and infectious upon subsequent exposure, but may suffer reproductive or developmental costs stemming from the physiological costs of maintaining a primed immune response. Conflating *infected* and *infectious* states, however, obscures the impact of priming on the correlative and dynamical relationships (Day, 2003; Berenos et al., 2009) between parasite replication, pathology, and transmission.

In directly transmitted infections, for example, parasite replication tends to be correlated with transmission, while pathology is often a byproduct of exploitation rather than a means of securing transmission (Roode et al., 2008). In fact, excessive pathology may curtail transmission by killing the host. In cases where peak pathology and peak transmission exhibit a time lag, an SEI (Susceptible-Exposed-Infectious)-type framework can be adapted to reflect different host fitness costs in infected and infectious states by allowing the latent “Exposed” compartment to experience pathology and disease-induced mortality. However, many pathogens of insects are obligate killers (Ebert and Weisser, 1997), meaning that they must kill the host in order to achieve transmission. There, the infected and infectious states are entirely separate, and pathology exerts a binary response on transmission (killing the host or not), although parasite replication will quantitatively affect transmission rates (Raymond et al., 2009) in hosts that do succumb.

While priming is often modeled in the context of direct transmission (Tate and Rudolf, 2012; Tidbury et al., 2012; Best et al., 2013), many microbes against which priming has been successfully demonstrated, including *Bacillus thuringiensis* in beetles (Roth et al., 2010; Milutinović et al., 2013; Tate and Graham, 2015), *Paenibacillus larvae* in bees (Hernández López et al., 2014), and baculoviruses in moths (Tidbury et al., 2010), exhibit obligate killer dynamics in nature (Tate, 2016). To illustrate the impact of transmission mode on the stability of a disease-free equilibrium (DFE) in insect populations in the context of parameters that might reflect priming, we can consider an analytically tractable model of three ordinary differential equations:

(1) $$\begin{array}{rcl} \frac{dS}{dt} & = & \theta -\mu S-\beta_{1}SI-\;\beta_{2}SD+\;\gamma I \end{array}$$

(2) $$\begin{array}{rcl} \frac{dI}{dt} & = & \;\beta_{1}SI+\;\beta_{2}SD-\alpha I-\gamma I \end{array}$$

(3) $$\begin{array}{rcl} \frac{dD}{dt} & = & \;\alpha I-\delta D \end{array}$$

Where *S* represents susceptible individuals, *I* is infected (and possibly infectious, if β_1_ > 0) individuals, and *D* represents individuals who have died and are possibly infectious (if β_2_ > 0; Tate, 2016). In directly transmitted infections, β_1_ > 0 and β_2_ is assumed to be 0, while in obligate killer transmission systems, β_2_ > 0 and β_1_ is 0. There are constant rates of birth θ, background mortality μ, disease-induced mortality α, recovery γ, and δ, which represents the decay (Fuller et al., 2012) of infectious cadavers.

The local asymptotic stability of the DFE in this system is conceptually similar to R0 (Diekmann and Heesterbeek, 2000). To analyze the DFE stability, we assume that all individuals N in the population are susceptible (disease-free), and calculate the eigenvalues of the Jacobian of this system evaluated at state variable values (*S* = *N*, 0, 0), for both βi conditions. We can then determine the parameter conditions that yield all negative eigenvalues and indicate stability of the DFE. If all eigenvalues do not have real negative values, this implies that the DFE is not stable and that disease could persist in the population.

For a direct transmission system, the DFE will be stable if

(4) $$\begin{array}{r} \frac{\beta_{1}N}{\gamma +\alpha }<1 \end{array}$$

For an obligate killer transmission system, the DFE will be stable if

(5) $$\begin{array}{r} \frac{\sqrt{\alpha \left(\alpha -2\delta +2\gamma +4\beta_{2}N\right)+{\left(\gamma -\delta \right)}^{2}}}{\left(\delta +\gamma +\alpha \right)}<1 \end{array}$$

The first (Equation 4) is easy to interpret; a lower transmission rate and higher recovery and disease-induced mortality rates will favor the stability of the DFE. Magnitude changes in recovery or disease-induced mortality contribute equally to the left-hand side of the term. This inequality aligns with standard R0 terms for SIS infections (Anderson and May, 1980). The DFE stability under obligate killer conditions (Equation 5), however, is less intuitive. The disease-induced mortality rate (α) exerts a bigger contribution in the numerator than the denominator, and cadaver decay (δ) always serves to decrease disease persistence. The role of recovery is less easily interpreted. The larger γ is relative to δ, the bigger its impact on the numerator, but also the bigger its impact on the denominator. Therefore, the decay rate of infectious cadavers is likely to alter the relative sensitivity of DFE stability to changes in disease induced mortality and recovery.

How could priming act on these parameters? If priming reduces susceptibility upon exposure or increases the rate of parasite killing by the host, we would expect that primed individuals would have slower parasite growth, reduced pathology, and lower transmission rates, assuming all of these were correlated. Therefore, as primed individuals recover and replace true susceptible individuals in the *S* compartment, β and α would both decrease over time. In direct transmission systems, we can interpret recovery γ as the rate of exit from infectiousness, which would increase. Therefore, the effect of priming on the stability of the DFE would depend on the magnitude change of β and γ relative to mortality (α). In obligate killer systems, the associated reduction in pathology should always reduce the force of infection.

The recovery parameter does demand special attention, however, since it will have different definitions depending on whether we are thinking in terms of host fitness or parasite replication. From a host life history point of view, recovery might refer to the end of pathology and an escape from the possibility of disease-induced mortality. Recovered individuals will transition into susceptible or primed populations, with corresponding reproductive rates rather than infected reproductive rates (Tate and Rudolf, 2012). But does symptom recovery terminate infectiousness? In an obligate killer system, it does, unless the parasites can persist for another opportunity at replication. In a direct transmission system, there is still the opportunity for parasites to be shed asymptomatically. This will be especially apparent when transmission stages are less virulent than replication stages, or when host tolerance mechanisms resolve the pathology (Medzhitov et al., 2012) but do not contribute to resistance. On the other hand, if recovery refers to an exit from infectiousness, then transmission may end but the host may continue to bear a cost to fitness initiated by pathology, or the physiological cost of immunity (Povey et al., 2009). Does priming improve recovery by increasing tolerance or by eliminating parasites? Are some types of priming mechanisms likely to be more costly than others? Resolving what it means to be a recovered individual will be crucial for linking the physiological and evolutionary costs of priming to the costs of infection and disease dynamics.

If we want to understand the interaction of priming and life history trade-offs, we should design experiments to evaluate how priming affects the transitions into and out of the *infected* state. If we want to understand the impact of priming on epidemics and microbial evolution, we need to think about how priming affects the transitions into and out of the *infectious* state. Ideally, we would evaluate both sides of the coin since there are likely to be ecological and evolutionary feedbacks between the costs of priming, microbial evolution, and disease dynamics. Here, I outline experimental parameters that will be useful for resolving ambiguity between infected and infectious states in priming systems.

**Natural history of the parasite:** Is it directly transmitted or obligate killer (or something else, like vertically- or vector-transmitted)? Is disease-induced mortality associated with microbial virulence, or immunopathology, or both? Finally, what is the probability of exposure (and re-exposure) for a given host to the microbe in a natural environment (Tate, 2016)? This last question is especially important for coupling epidemiological dynamics with the evolutionary maintenance of priming.
**Disease-induced mortality rates:** This parameter contributes to the transition out of infected and infectious states. Fortunately, this is among the most commonly measured parameters in immune priming experiments (e.g., Pham et al., 2007; Roth et al., 2010; Hernández López et al., 2014; Tate and Graham, 2015). However, dose-response curves would fortify the distribution of rate estimates (Ben-Ami et al., 2010) related to immune priming.
**Microbe load over the infection period:** Microbe load is an important underlying driver of many epidemiological parameters, as it often contributes to both recovery and disease-induced mortality. Measuring microbe load would help to determine whether priming acts through resistance, tolerance, or both (Medzhitov et al., 2012). Despite this, the temporal dynamics of parasite growth and killing are rarely measured in priming studies.
**Duration of infectiousness:** How many susceptible individuals can a parasitized individual infect per unit time? We can use this data to estimate the temporal distribution of transmissibility (Fuller et al., 2012) for primed and unprimed hosts, and to figure out when infected individuals are also infectious. In obligate killer systems, priming should always decrease infectiousness, but it is unknown whether priming reduces transmissibility in those who do succumb. This is likely to depend on how priming interferes with microbial growth and life history.
**Dose and virulence response curves:** Both parasite dose and virulence could impact the efficacy of priming, through threshold responses for memory induction, damage-induced memory, or costs associated with virulence that interfere with production of memory. Are shifts in parameters between primed and unprimed individuals constant across dose, or is there an interaction with dose (Ben-Ami et al., 2010)? If the latter, what is the distribution of new doses stemming from infectious individuals? How “perfect” is priming, in terms of preventing future infections—does it simply increase the dose needed to colonize the host, or does it protect against the full range of doses that a host is likely to encounter in the wild (Tate, 2016)? Along similar lines, how does parasite virulence correlate with the induction of immune memory?

Resolving the conflation of infected and infectious states will allow us to more accurately predict the impact of insect immune priming on both life history evolution and disease dynamics. The illustrative analysis above serves solely to highlight parameters that could be influenced by immune priming. Future studies should consider the relationship between infection and infectiousness when explicitly incorporating immune priming into models of disease dynamics. By measuring sensible parameters that link within- and between-host dynamics, future empirical studies could help resolve the ambiguity surrounding the dependence of disease dynamics on the interaction between immune priming and transmission mode.

## Author contributions

AT performed all functions related to preparing this manuscript.

## Funding

AT is supported by the Food Research Initiative Competitive Grant No. 2014-67012-22278 from the USDA National Institute of Food and Agriculture.

### Conflict of interest statement

The author declares that the research was conducted in the absence of any commercial or financial relationships that could be construed as a potential conflict of interest.

## References

[B1] Anderson R. M. May R. M. (1980). Infectious diseases and population cycles of forest insects. Science 210, 658–661. 10.1126/science.210.4470.658 17815156

[B2] Ben-Ami F. Ebert D. Regoes R. R. (2010). Pathogen dose infectivity curves as a method to analyze the distribution of host susceptibility: a quantitative assessment of maternal effects after food stress and pathogen exposure. Am. Nat. 175, 106–115. 10.1086/648672 19911987

[B3] Berenos C. Schmid-Hempel P. Mathias Wegner K. (2009). Evolution of host resistance and trade-offs between virulence and transmission potential in an obligately killing parasite. J. Evol. Biol. 22, 2049–2056. 10.1111/j.1420-9101.2009.01821.x 19732263

[B4] Best A. Tidbury H. White A. Boots M. (2013). The evolutionary dynamics of within-generation immune priming in invertebrate hosts. J. R. Soc. Interface 10:20120887. 10.1098/rsif.2012.0887 23269850 PMC3565738

[B5] Day T. (2003). Virulence evolution and the timing of disease life-history events. Trends Ecol. Evol. 18, 113–118. 10.1016/S0169-5347(02)00049-6

[B6] Diekmann O. Heesterbeek J. (2000). Mathematical Epidemiology of Infectious Diseases: Model Building, Analysis and Interpretation. Chichester: John Wiley.

[B7] Ebert D. Weisser W. W. (1997). Optimal killing for obligate killers: the evolution of life histories and virulence of semelparous parasites. Proc. R. Soc. Lond. B Biol. Sci. 264, 985–991. 10.1098/rspb.1997.0136 9263465 PMC1688549

[B8] Fuller E. Elderd B. D. Dwyer G. (2012). Pathogen persistence in the environment and insect-baculovirus interactions: disease-density thresholds, epidemic burnout and insect outbreaks. Am. Nat. 179, E70–E96. 10.1086/664488 22322229 PMC3814039

[B9] Hernández López J. Schuehly W. Crailsheim K. Riessberger-Gallé U. (2014). Trans-generational immune priming in honeybees. Proc. R. Soc. B Biol. Sci. 281:20140454. 10.1098/rspb.2014.0454 24789904 PMC4024302

[B10] Medzhitov R. Schneider D. S. Soares M. P. (2012). Disease tolerance as a defense strategy. Science 335, 936–941. 10.1126/science.1214935 22363001 PMC3564547

[B11] Milutinović B. Fritzlar S. Kurtz J. (2013). Increased survival in the red flour beetle after oral priming with bacteria-conditioned media. J. Innate Immun. 6, 306–314. 10.1159/000355211 24216503 PMC6742957

[B12] Moret Y. (2006). “Trans-generational immune priming”: specific enhancement of the antimicrobial immune response in the mealworm beetle, *Tenebrio molitor*. Proc. R. Soc. B Biol. Sci. 273, 1399–1405. 10.1098/rspb.2006.3465 16777729 PMC1560290

[B13] Pham L. N. Dionne M. S. Shirasu-Hiza M. Schneider D. S. (2007). A specific primed immune response in *Drosophila* is dependent on phagocytes. PLoS Pathog. 3:e26. 10.1371/journal.ppat.0030026 17352533 PMC1817657

[B14] Povey S. Cotter S. C. Simpson S. J. Lee K. P. Wilson K. (2009). Can the protein costs of bacterial resistance be offset by altered feeding behaviour? J. Anim. Ecol. 78, 437–446. 10.1111/j.1365-2656.2008.01499.x 19021780

[B15] Raymond B. Ellis R. J. Bonsall M. B. (2009). Moderation of pathogen-induced mortality: the role of density in *Bacillus thuringiensis* virulence. Biol. Lett. 5, 218–220. 10.1098/rsbl.2008.0610 19033132 PMC2665808

[B16] Roode J. C. D. Yates A. J. Altizer S. (2008). Virulence-transmission trade-offs and population divergence in virulence in a naturally occurring butterfly parasite. Proc. Natl. Acad. Sci. U.S.A. 105, 7489–7494. 10.1073/pnas.0710909105 18492806 PMC2396697

[B17] Roth O. Joop G. Eggert H. Hilbert J. Daniel J. Schmid-Hempel P.. (2010). Paternally derived immune priming for offspring in the red flour beetle, *Tribolium castaneum*. J. Anim. Ecol. 79, 403–413. 10.1111/j.1365-2656.2009.01617.x 19840170

[B18] Tate A. T. (2016). A general model for the influence of immune priming on disease prevalence. Oikos. 10.1111/oik.03274. [Epub ahead of print].

[B19] Tate A. T. Graham A. L. (2015). Trans-generational priming of resistance in wild flour beetles reflects the primed phenotypes of laboratory populations and is inhibited by co-infection with a common parasite. Funct. Ecol. 29, 1059–1069. 10.1111/1365-2435.12411

[B20] Tate A. T. Rudolf V. H. W. (2012). Impact of life stage specific immune priming on invertebrate disease dynamics. Oikos 121, 1083–1092. 10.1111/j.1600-0706.2011.19725.x

[B21] Tidbury H. J. Best A. Boots M. (2012). The epidemiological consequences of immune priming. Proc. R. Soc. B Biol. Sci. 279, 4505–4512. 10.1098/rspb.2012.1841 22977154 PMC3479815

[B22] Tidbury H. J. Pedersen A. B. Boots M. (2010). Within and transgenerational immune priming in an insect to a DNA virus. Proc. R. Soc. B Biol. Sci. 278, 871–876. 10.1098/rspb.2010.1517 20861049 PMC3049047

